# Stigma as a Barrier to Participant Recruitment of Minority Populations in Diabetes Research: Development of a Community-Centered Recruitment Approach

**DOI:** 10.2196/26965

**Published:** 2021-05-03

**Authors:** Suzanne Mitchell, Alexa Bragg, Ioana Moldovan, Shakiyla Woods, Katherine Melo, Jessica Martin-Howard, Paula Gardiner

**Affiliations:** 1 Boston Medical Center Boston, MA United States; 2 Department of Family Medicine Boston University School of Medicine Boston, MA United States; 3 Department of Family Medicine and Community Health University of Massachusetts Medical School Worcester, MA United States

**Keywords:** diabetes, stigma, research, recruitment, minority health, disparities, virtual health, virtual management

## Abstract

**Background:**

The development of evidence-based care geared towards Black and Latina women living with uncontrolled type 2 diabetes is contingent upon their active recruitment into clinical interventions. Well-documented impediments to recruitment include a historical mistrust of the research community and socioeconomic factors that limit awareness and access to research studies. Although sociocultural and socioeconomic factors deter minorities from participating in clinical research, it is equally important to consider the role of stigma in chronic disease intervention studies.

**Objective:**

We aim to share our discovery of diabetes-related stigma as an underrecognized impediment to recruitment for the Women in Control 2.0 virtual diabetes self-management education study.

**Methods:**

Our initial recruitment plan used traditional strategies to recruit minority women with uncontrolled type 2 diabetes, which included letters and phone calls to targeted patients, referrals from clinicians, and posted flyers. After engaging a patient advisory group and consulting with experts in community advocacy, diabetes-related stigma emerged as a prominent barrier to recruitment. The study team reviewed and revised recruitment scripts and outreach material in order to better align with the lived experience and needs of potential enrollees.

**Results:**

Using a more nuanced, community-centered recruitment approach, we achieved our target recruitment goal, enrolling 309 participants into the study, exceeding our target of 212.

**Conclusions:**

There is a need for updated recruitment methods that can increase research participation of patients who experience internalized diabetes stigma. To address disparities in minority health, further research is needed to better understand diabetes-related stigma and devise strategies to avert or address it.

## Introduction

Type 2 diabetes mellitus (T2DM) disproportionately impacts racial and ethnic minority communities in the United States [1]. Although there is a need for research in delivering evidence-based care tailored to a diversity of patients living with diabetes, minority persons represented only 36.1% of individuals enrolled in clinical trials sponsored by the National Institutes of Health in 2018 [2]. Well-documented impediments to recruitment of minorities for research trials are linked to socioeconomic factors and mistrust of the research community stemming from structural racism [3-9]. Included in this viewpoint is the experience of diabetes-related stigma among minority populations as a barrier to recruitment. Diabetes-related stigma is defined as “the experiences of negative feelings such as exclusion, rejection, or blame due to the perceived stigmatization of having diabetes” [10].

Our research team encountered these challenges in the recruitment of Black and Latina women for Women in Control 2.0 (WIC2), a randomized controlled trial (NCT02726425) studying the comparative effectiveness of online diabetes self-management (DSM) medical group visit (MGV) education with in-person DSM MGV education. However, it was the participants’ experience of diabetes-related stigma, a persistent and underrecognized barrier to recruitment, that proved to be our greatest challenge in reaching our enrollment target of 212 study participants completing 6 out of 8 group visits. Herein, we share our lessons learned from our encounter with the issue of diabetes-related stigma while recruiting minority women with T2DM in clinical research.

WIC2 is a 2-arm randomized controlled trial (National Institute of Diabetes and Digestive and Kidney Diseases; grant no. R01DK106531) comparing the effectiveness of DSM MGV education delivered in a virtual social-gaming platform (intervention) or a face-to-face setting (control) [11]. We aimed to enroll 212 English- and Spanish-speaking Black and Latina women with uncontrolled diabetes (baseline hemoglobin A_1c_ [HbA_1c_] ≥ 8.0% or 64 mmol/mol) from the Boston Medical Center Health System over the course of a 4-year period. Participants were assigned into 13 cohorts (8-10 participants each) of virtual and face-to face MGVs. The 2 primary outcomes are mean changes in pre-to-post DSM MGV physical activity and glucose control between baseline and 6-month follow-up. Prior to randomization, we obtained written informed consent from all participants. During the 8-week MGVs, participants engaged in facilitated discussions adapted from the Power to Prevent curriculum created by the Center for Disease Control and Prevention’s National Diabetes Education Program, which emphasizes lifestyle changes including healthy eating and physical activity [12]. Participants also received a brief individual clinical consultation at each session to discuss medical treatments, gaps in care, and ensure safety.

## Methods

### Initial Recruitment Protocol (November 2016 to August 2017)

Prior to the start of the clinical trial, we engaged a patient advisory group (PAG) comprising 10 Black women with T2DM from the community to review and inform our recruitment strategy and study materials. The PAG met for 10 sessions and provided feedback on our outreach protocol and scripts. In addition, to prepare for recruitment, we established channels for clinician referrals at Boston Medical Center and protocols for direct outreach by study staff. To encourage referrals from allied health providers, the WIC2 principal investigator (SM) announced the study opportunity at local community health centers and during hospital grand rounds. Outreach coordinators distributed study flyers at affiliated clinics and community health centers to raise awareness about the study. The team also identified eligible potential participants by using electronic medical record (EMR) query; these patients received a study recruitment letter followed by a telephone call by a trained research assistant.

### Challenges Encountered

Despite initial success with recruitment for the first 3 study cohorts, after enrolling 46 participants, we were unable to reach our enrollment milestones. Between August 2017 and October 2017, we were unable to enroll the required 16 participants to conduct the fourth cohort of study implementation. Careful assessment of our recruitment channels and procedures revealed key deficits in our strategy. First, we were not successful with clinician referrals to the study. Feedback from primary care colleagues revealed that many providers were too busy to explain the study opportunity to patients, while others raised concerns that study clinicians would alter patients’ medication regimens without consulting the primary providers. Despite our attempts to reassure clinicians, referrals from clinics were low. As an alternative means of recruiting participants, we attempted to foster community partnerships using strategies from community-based participatory research, such as outreach to local churches and businesses, including beauty salons, grocery stores, and laundromats in target communities [13]. However, similar to challenges encountered by other researchers, our efforts to establish and build sustainable community partnerships were stymied by time and resource constraints set by our study timeline and budget [14,15]. We found that potential community partners were themselves strapped for resources, and generating interest in our research effort was difficult.

### Re-evaluation of the Recruitment Protocol (August 2017 to December 2017)

We investigated the failure modes in our recruitment approach by consulting with community health advocacy experts experienced in working with vulnerable populations, re-engaging our PAG, and observing study staff conversations with potentially eligible participants to identify reasons for declination. We first conducted interviews with local public health activists with extensive experience working with the Black and Latino Boston-area communities to explore reasons for participation resistance. They advised us to consider the role of diabetes-related stigma: the sense of shame associated with being responsible for developing or failing to manage a disease or illness [16].

In order to broaden our understanding of potential barriers to participation, we reconvened the WIC2 PAG to review additional recruitment materials, outreach scripts used by study staff, and elements of the study website. It was evident during the reunion that our recruitment materials did not fundamentally address experiences of isolation and disempowerment. It became apparent after observing recruitment outreach calls that our recruitment script underemphasized the study as an opportunity to participate in a relationship-centered intervention and instead focused on the transactional, compensatory components of the study.

### Revised Recruitment Protocol (December 2017 to October 2019)

Starting December 2017, we hired culturally concordant staff with previous outreach experience from the local Boston community to deliver a relationship-centered message during recruitment. By drawing upon a growing understanding of the existence and implications of stigma, we implemented four key strategies to revise our recruitment approach: (1) communicate study relevance to the community, (2) promote the idea of personal empowerment with message mapping, (3) cultivate connections between participants prior to the start of the DSM MGVs, and (4) encourage enrollees to recruit others via snowball sampling. Each of these strategies helped us engage women who may have otherwise been unwilling to participate in clinical research due to diabetes-related stigma.

### Study Relevance to the Community

When introducing the WIC2, we emphasized our mission for health equity and educated potential participants about how the DSM MGVs could potentially reduce disparities in diabetes care. To lessen the sense of isolation often associated with stigmatizing illnesses, we shared facts regarding high rates of diabetes among communities of color. Relaying information in a way that reminded potential participants that their involvement mattered beyond the study-specific aims was crucial. We also highlighted the fact that their active engagement embodied the power of a community to effect change. We emphasized the potential of giving back to the community in order to create a lasting dialogue around diabetes care management and prevention for families at risk.

### Personal Empowerment With Message Mapping

Informed by valuable insights gained from our PAG collaborators, flyers were redesigned to capture three key messages of the study: taking control of diabetes, experiencing personal transformation, and finding a community. We also recorded testimonial videos featuring PAG participants who shared their experience working with the study team and the WIC2 intervention. These testimonials, which were posted on the newly designed WIC2 website, framed the study as an opportunity for self-empowerment through engagement in a strengths-based DSM education intervention.

### Cultivating Connections Among Current Participants

Potential participants were invited to attend enrollment sessions in groups of 6 to 10 to mirror the upcoming group-based format during the intervention. The purpose of these informal sessions was to foster a sense of connection and familiarity among participants before the official start of the study; they were encouraged to introduce themselves and share their lived experience with T2DM. In addition, reminder calls and appointment cards were issued a few days prior to enrollment to maintain interest and excitement about future participation in the study.

### Involving Former Participants in Recruitment

We found that women who had themselves participated in the study were excellent advocates for study participation. Because of their goodwill toward WIC, we implemented a Refer-a-Friend program, whereby we offered current and past participants US $20 for every friend referred who ultimately enrolled in the study and attended at least one group visit. By the end of the study period, 58 participants were recruited by word of mouth or participant referral. The referral program also increased participants’ sense of giving back by contributing to the WIC2 recruitment mission.

## Results

**Figure 1:**
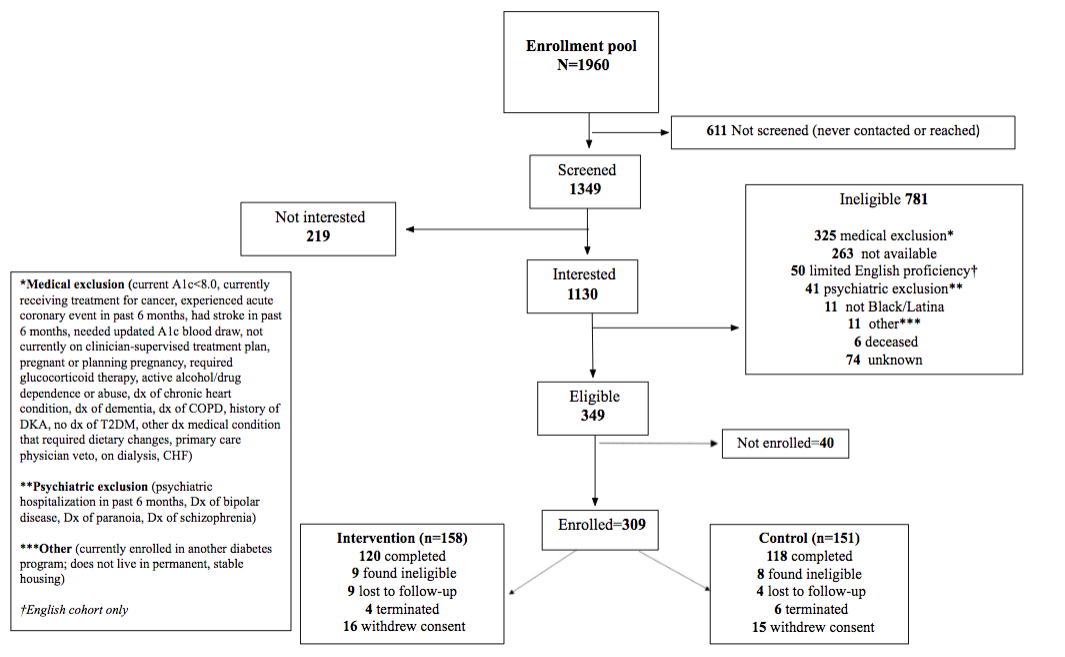
Women in Control 2.0 Consolidated Standards of Reporting Trials (CONSORT) diagram. CHF: congestive heart failure; COPD: chronic obstructive pulmonary disease; DKA: diabetic ketoacidosis; dx: diagnosed with; T2DM: type 2 diabetes mellitus.

A total of 1960 patients were identified as potentially eligible for the WIC2 study from a variety of sources including EMR query, flyers, word of mouth, and referrals. After completing the 17th study cohort in December 2019, 1349 potential participants were screened, 349 were eligible, and 309 were enrolled (Figure 1).

Of the 1449 potentially eligible English-language patients, 88.47% (1282/1449) were recruited by EMR query, 5.45% (79/1449) by flyers, 4.00% (58/1449) by word of mouth or participant referral, 1.38% (20/1449) by primary care provider referral, and 0.69% (10/1449) by other sources. All 511 Spanish-speaking participants were identified and recruited via EMR query. Despite the low rate of eligibility, of the participants who were deemed eligible, 88.5% (309/349) enrolled, 90.9 % (281/309) completed baseline HbA_1c_ and/or physical activity data collection, and 73.1% (207/281) attended at least 6 out of 8 group visits. Participants who completed baseline requirements had a mean age of 55 years (SD 10), a mean baseline HbA_1c_ of 9.9% (SD 1.8), and 69.7% reported Medicaid or Medicare as their primary insurance provider. The characteristics of participants who completed baseline activities (N=281) are displayed in Table 1.

Common reasons for ineligibility included unavailability to attend MGVs at scheduled times, current HbA_1c_<8.0%, outdated HbA_1c_ (outside of the 90-day window prior to the start of the first MGV), and lack of interest in participating.

Qualitative analysis of transcripts from 3 focus groups (n=22) that were conducted postintervention elucidated feelings of shame and responsibility for lack of “control” of their T2DM. Participants describe a sense of shame that diabetes progression is self-caused, which translated to diabetes-related stigma.

[Women in Control] *has helped me elevate my self-esteem and to walk because before I wanted to be locked away in my home without doing anything, a strong depression...in my house, I am alone with my husband, like that for me it's harder. But...Women in control…activated me.*

Virtual World participant

And I tell you, my children offer me because of course they are not sick. They tell me, “mama, eat,” and I say, “no, son, you guys eat and know how to eat because if one day you are sick, do not blame me because a lot of people say it's hereditary.” But that's a lie, we give it to ourselves because of our carelessness. [Background laughter] Because I'm going to tell you something, my mom, my dad and my brothers never had sugar and only I have it. So that was because of my carelessness.

face-to-face participant

This qualitative data supports the insights gleaned from the PAG and public health activists between December 2017 and October 2019.

**Table 1:** Baseline sample characteristics (N=281).

Characteristic		Total (N=281, 100%)	Control (n=137, 49%)^a^	Intervention (n=144, 51%)^a^
Age (years), mean (SD)		56 (10)	55 (10)	56 (11)
**Ethnicity, n (%)**				
	Hispanic/Latina	98 (34.9)	46 (33.6)	52 (36.1)
	White	25 (8.9)	13 (9.5)	12 (8.3)
**Race, n (%)**				
	Black	185 (65.8)	92 (67.2)	93 (64.6)
	Other	60 (21.4)	27 (19.7)	33 (22.9)
	Refused to answer/unknown	1 (0.4)	0 (0.0)	1 (0.7)
	More than one race	6 (2.1)	2 (1.5)	4 (2.8)
Spanish speaking, n (%)		53 (18.9)	26 (19.0)	27 (18.6)
**Insurance, n (%)**				
	Medicaid (MassHealth)	129 (45.9)	64 (47.4)	65 (44.4)
	Medicare	31 (11.0)	17 (12.4)	14 (9.7)
	Medicaid + Medicare	46 (16.4)	21 (15.3)	25 (17.4)
	Commercial	64 (22.8)	29 (21.2)	35 (24.3)
	Don’t know or prefer not to answer	7 (2.5)	3 (2.2)	4 (2.8)
Has children, n (%)		249 (88.6)	121 (88.3)	128 (89.9)
**Work status, n (%)**				
	Full-time	69 (24.6)	33 (24.1)	36 (25.0)
	Retired	26 (9.3)	13 (9.5)	13 (9.0)
	Disabled	61 (21.7)	29 (21.2)	32 (22.2)
	Part-time	39 (13.9)	22 (16.1)	17 (11.8)
	Unemployed	34 (12.1)	16 (11.7)	18 (12.5)
	Other	12 (4.3)	6 (4.4)	6 (4.2)
	Refused to answer/unknown	13 (4.6)	5 (3.6)	8 (5.6)
**Education history, n (%)**				
	Less than high school (grade 0-8)	52 (18.5)	25 (18.4)	27 (18.6)
	Some high school (grade 9-<12)	36 (12.8)	21 (15.3)	15 (10.4)
	GED^b^ + high school graduate	53 (18.8)	23	30
	Post high school	139 (49.5)	68 (49.6)	71 (49.3)
	Refused to answer/unknown	1 (0.4)	0 (0.0)	1 (0.7)
Attended at least 6 group visits, n (%)		207 (73.7)	99 (72.3)	108 (75.0)
Hemoglobin A_1c_ (%), mean (SD)		10.0 (2.0)	10.0 (2.0)	10.0 (2.0)

^a^Percentages below this heading are based on the total for this column only (column %).

^b^GED: General Educational Development Test.

## Discussion

Although sociocultural and socioeconomic factors deter minority persons from enrolling in clinical research studies, we also encountered diabetes-related stigma as an unseen barrier to recruitment. Similar findings regarding the impact of disease-related stigma on research efforts are reported in other research fields including HIV and mental health research [17-19]. Despite diabetes-related stigma being potentially underrecognized among researchers, it is a common shared experience among those living with diabetes [20]. The WIC2 study participants in our postintervention focus groups indicated that, in their communities, people with T2DM are often depicted with negative traits, such as “laziness” and a lack of self-control, resulting in a desire not to disclose the fact that one is living with diabetes. Myriad testimonies from individuals living with diabetes reveal a shared sense of personal shame related to feeling “responsible” for acquiring diabetes [15]. Stigmatization of T2DM as a lifestyle disease contributes to a sense of “hopelessness and fear of discussing diabetes complications” and bars patients from seeking clinical care [21]. As a result, many patients have poor diabetes control and health outcomes [16,22,23]. We redesigned our recruitment strategy to focus on relationship-centered communication, which addressed unseen diabetes-related stigma in a research context [23]. To accomplish this goal, we hired culturally concordant research staff who communicated the study’s relevance to the community, emphasized personal empowerment with message mapping, fostered connections among participants prior to the start of the DSM MGVs, and encouraged current and former participants to recruit others via snowball sampling. Collectively, these efforts helped us successfully avert the risk posed by diabetes-related stigma to our study implementation. Most importantly, we exercised cultural humility in the face of this unforeseen obstacle, allowing ourselves to be educated and navigated by our participant community and our colleagues toward a successful solution.

To effectively address disparities in health outcomes and healthcare, we must design and conduct health services research involving those most at risk for poor health outcomes. These efforts require a diverse pool of participants from underserved and underrepresented communities. Through WIC2, we identified diabetes-related stigma as an unseen impediment to participation in diabetes research. Although stigma has been noted in other diseases like HIV, there is limited research specifically about diabetes-associated stigma, especially its role as a barrier to recruitment in clinical trials. Further work is needed to better understand diabetes-related stigma and devise evidence-based strategies to avert or address it.

## Abbreviations

DSM: diabetes self-management
EMR: electronic medical record
HbA_1c_: hemoglobin A_1c_
MGV: medical group visit
PAG: patient advisory group
T2DM: type 2 diabetes mellitus
WIC2: Women in Control 2.0
